# Crystal structure of bis­{1-phenyl-3-methyl-4-[(quinolin-3-yl)imino­methyl-κ*N*]-1*H*-pyrazol-5-olato-κ*O*}zinc methanol 2.5-solvate from synchrotron X-ray diffraction

**DOI:** 10.1107/S2056989017010441

**Published:** 2017-07-18

**Authors:** Anatoliy S. Burlov, Valery G. Vlasenko, Pavel V. Dorovatovskii, Yan V. Zubavichus, Victor N. Khrustalev

**Affiliations:** a Institute of Physical and Organic Chemistry, Southern Federal University, 194/2 Stachki Ave., Rostov-on-Don 344090, Russian Federation; b Institute of Physics, Southern Federal University, 194 Stachki Ave., Rostov-on-Don 344090, Russian Federation; c National Research Centre ‘Kurchatov Institute’, 1 Acad. Kurchatov Sq., Moscow 123182, Russian Federation; d Inorganic Chemistry Department, Peoples’ Friendship University of Russia (RUDN University), 6 Miklukho-Maklay St., Moscow 117198, Russian Federation

**Keywords:** crystal structure, pyrazole-quinoline, Schiff base ligands, zinc complex, synchrotron radiation

## Abstract

The title compound, [Zn(C_20_H_15_N_4_O)_2_]·2.5CH_3_OH, **I**, was synthesized *via* the reaction of zinc acetate with the respective ligand and isolated as a methanol solvate, *i.e.*, as **I·2.5CH_3_OH**. The crystal structure is triclinic (space group *P*-1), with two complex mol­ecules (***A*** and ***B***) and five methanol solvent mol­ecules in the asymmetric unit. One of the five methanol solvent mol­ecules is disordered over two sets of sites, with an occupancy ratio of 0.75:0.25. Mol­ecules ***A*** and ***B*** are conformers and distinguished by the conformations of the bidentate 1-phenyl-3-methyl-4-[(quinolin-3-yl)imino­meth­yl]-1*H*-pyrazol-5-olate ligands. In both mol­ecules, the zinc cations have distorted tetra­hedral coordination spheres, binding the monoanionic ligands through the pyrazolo­late O and imine N atoms. The two ligands adopt slightly different conformations in terms of the orientation of the terminal phenyl and quinoline substituents with respect to the central pyrazolo­late moiety. The mol­ecular geometries of ***A*** and ***B*** are supported by intra­molecular C—H⋯O and C—H⋯N hydrogen bonds. In the crystal of **I**, mol­ecules form dimers both by secondary inter­molecular Zn⋯O [3.140 (2)–3.553 (3) Å] and π–π stacking inter­actions. The dimers are linked by inter­molecular hydrogen bonds through the solvent methanol mol­ecules into a three-dimensional network.

## Abstract 2

A synthetic approach to the new zinc complex based on amino­methyl­ene derivative of 1-phenyl-3-methyl-4-[(quinolyl-3-yl)imino­meth­yl]-1*H*-pyrazol-5(4*H*)-one and its structural characterization by synchrotron single-crystal X-ray diffraction are reported.

## Chemical context   

Zinc complexes of azomethine ligands with heterocyclic derivatives are the subject of significant inter­est owing to their 
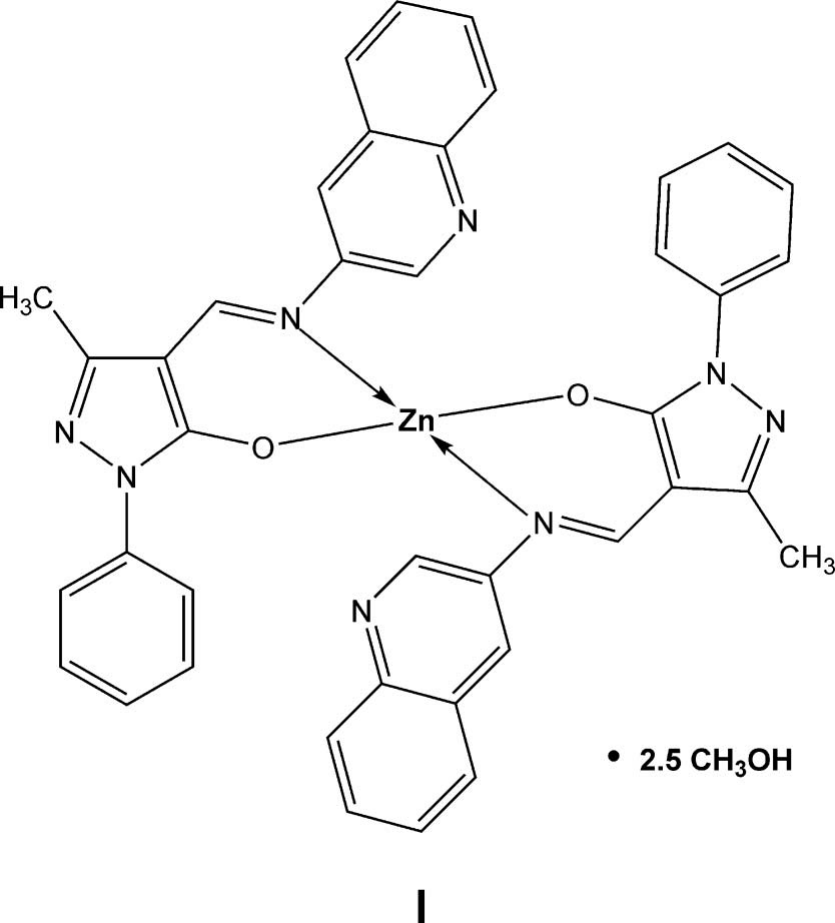
photo- (PL) and electro-luminescent (EL) properties (Burlov, Chesnokov *et al.*, 2014 ▸; Burlov, Koshchienko *et al.*, 2014 ▸; Burlov *et al.*, 2015 ▸, 2016 ▸; Nikolaevskii *et al.*, 2014 ▸). The thermal stability, high vitrification temperatures, easy sublimation during deposition of thin amorphous films, variability of structures, relative synthetic affordability and electron-transfer characteristics of such zinc complexes make them good candidates for application as active layers for organic light-emitting diode (OLED) devices.

We report here a synthetic approach for the preparation of a new zinc complex based on an amino­methyl­ene derivative of 1-phenyl-3-methyl-4-[(quinolin-3-yl)imino­meth­yl]-1*H*-pyra­zol-5(4*H*)-one and 3-amino­quinoline, and its structural characterization by synchrotron single-crystal X-ray diffraction.

## Structural commentary   

**Figure 1:**
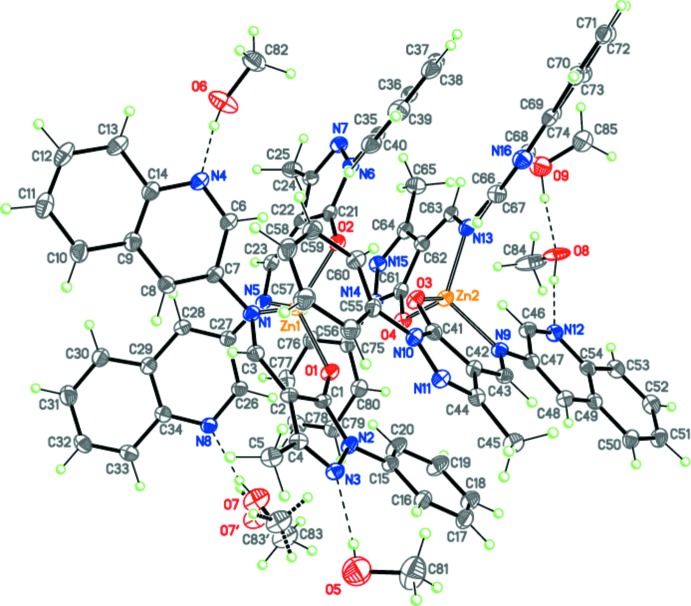
The structures of the mol­ecular entities in **I·2.5CH_3_OH**. Mol­ecules ***A*** and ***B*** are shown. Displacement ellipsoids are depicted at the 50% probability level. H atoms are presented as small spheres of arbitrary radius. Dashed lines indicate inter­molecular O—H⋯N hydrogen bonds.

Compound **I**, [Zn(C_20_H_15_N_4_O)_2_], crystallizes in the triclinic space group *P* 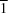 with two complex mol­ecules (***A*** and ***B***) and five methanol solvent mol­ecules in the asymmetric unit, *i.e.*, as **I·2.5CH_3_OH**, with one of the five methanol solvent mol­ecules being disordered over two positions in a 0.75:0.25 ratio (Fig. 1 ▸). Complex mol­ecules ***A*** and ***B*** are conformers and distinguished by the conformations of the bidentate 1-phenyl-3-methyl-4-[(quino­lin-3-yl)imino­meth­yl]-1*H*-pyrazol-5-olate ligands.

The zinc cations of ***A*** and ***B*** in **I** are four-coordinated by two monoanionic *O*,*N*-chelating ligands, which bind to the cation through pyrazolo­late O and imine N atoms. The coordination sphere around each zinc cation can be described as distorted tetra­hedral [the bond-angle ranges are 94.83 (8)–121.00 (8) and 95.73 (8)–118.36 (10)° for mol­ecules ***A*** and ***B***, respectively], with dihedral angles between the planar six-membered chelating rings (r.m.s. deviations are 0.031/0.021 and 0.017/0.033 Å for mol­ecules ***A*** and ***B***, respectively) of 82.97 (7) and 84.52 (7)° for mol­ecules ***A*** and ***B***, respectively.

**Figure 2:**
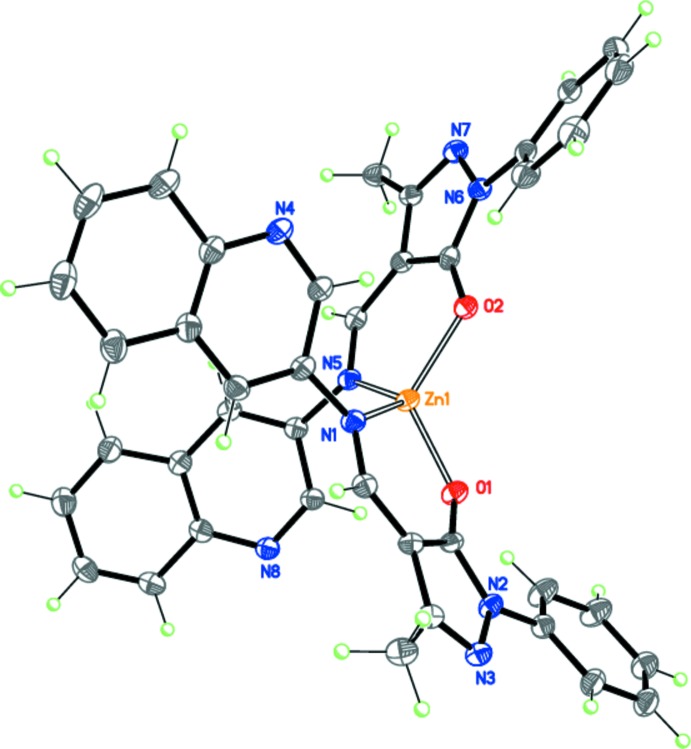
The mol­ecular structure of conformer ***A***.

**Figure 3:**
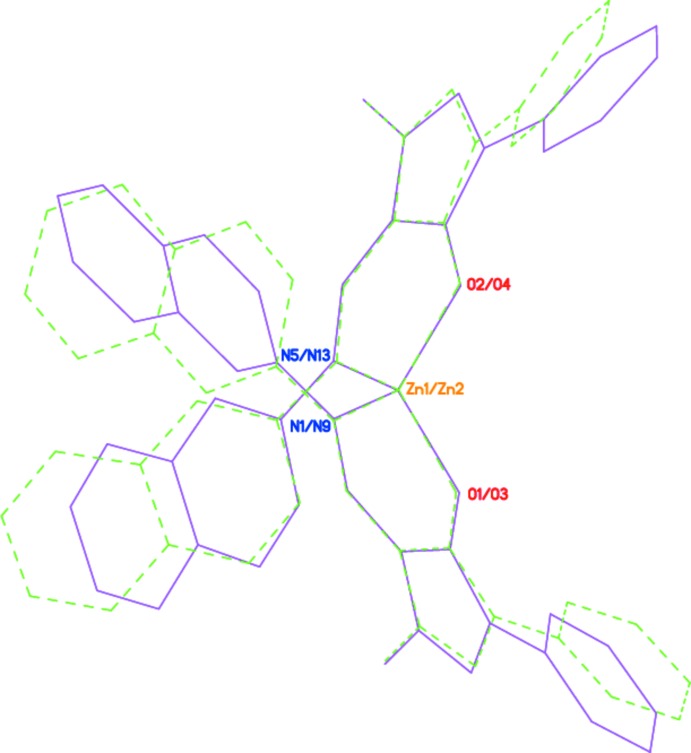
Comparison of the geometries of conformers ***A*** (magenta) and ***B*** (green dashed lines).

The four pyrazolo­late ligands in mol­ecules ***A*** and ***B*** of **I** adopt different conformations. The main difference pertains to the twist angles of the terminal phenyl and quinoline substituents relative to the central imino­methyl-1*H*-pyrazol-5-olate fragment. In mol­ecule ***A*** (Fig. 2 ▸), the corresponding angles are 20.40 (13) and 25.34 (8)° for the phenyl groups, and 37.02 (5) and 52.57 (7)° for the quinoline substituents, whereas in mol­ecule ***B***, these angles are 15.03 (13) and 8.24 (11)° for the phenyl groups, and 27.47 (10) and 26.08 (6)° for the quinoline substituents. Thus, one of the two 1-phenyl-3-methyl-4-[(quinolin-3-yl)imino­meth­yl]-1*H*-pyrazol-5-olate ligands in mol­ecule ***B*** is flattened, while one of the two pyrazolo­late ligands in mol­ecule ***A*** is substanti­ally twisted (Fig. 3 ▸). The mol­ecular conformation observed for **I** is supported by weak intra­molecular hydrogen bonds: C6—H6⋯O2 in mol­ecule ***A*** and C66—H66⋯O3 and C46—H46⋯N13 in mol­ecule ***B*** (Table 1 ▸).

## Supra­molecular features   

**Figure 4:**
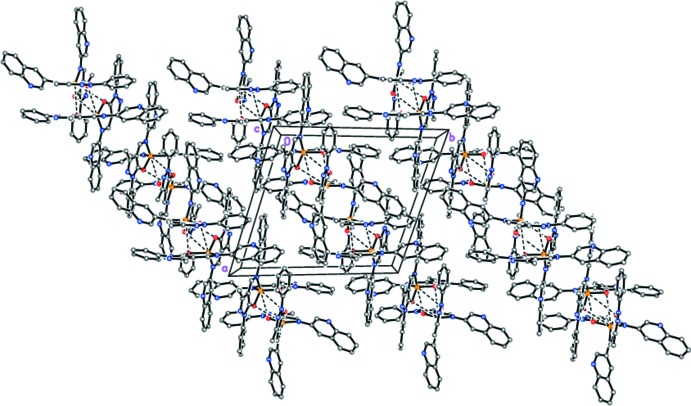
The crystal packing of the dimers present in **I**. Dashed lines indicate inter­molecular secondary Zn⋯O inter­actions.

In the crystal of **I**, mol­ecules form robust dimers both by inter­molecular secondary Zn⋯O inter­actions [Zn1⋯O3 = 3.386 (2) Å, Zn1⋯O4 = 3.279 (3) Å, Zn2⋯O1 = 3.553 (3) Å and Zn2⋯O2 = 3.140 (2) Å] and π–π stacking inter­actions between the O2/N5/N6/N7/C21–C24 and O4/N13/N14/N15/C61–C64 imino-methyl-pyrazolo­nate fragments {the shortest distances are N6⋯C63 [3.083 (3) Å], C21⋯C62 [3.210 (4) Å], C24⋯C64 [3.216 (4) Å], C21⋯C61 [3.261 (3) Å], N14⋯C23 [3.293 (4) Å], C22⋯C61 [3.297 (4) Å], N6⋯C62 [3.319 (3) Å] and N14⋯C22 [3.362 (3) Å]}, as well as phenyl and pyridine rings [the *C*g1⋯*C*g2 distance is 3.330 (6) Å, where *C*g1 is the centroid of the C35–C40 phenyl ring and *C*g2 is the centroid of the N16/C66–C69/C74 pyridine ring] (Fig. 4 ▸). The dimers are bound to each other by inter­molecular C—H⋯π hydrogen bonds [the strongest is C17—H17⋯*C*g3^vii^ (H⋯*C*g3^vii^ = 2.48 Å and C—H⋯*C*g3^vii^ = 169°), where *C*g3^vii^ is the centroid of the C69^vii^–C74^vii^ benzene ring; symmetry code: (vii) −*x*, −*y*, −*z* + 1] and π–π stacking inter­actions {the shortest distances are between the C75–C80 and C75^viii^–C80^viii^ phenyl rings [C75⋯C79^viii^ = 3.196 (4) Å and C80⋯C80^viii^ = 3.279 (4) Å]; symmetry code: (viii) −*x*, −*y* + 1, −*z* + 1}, as well as C—H⋯O and N⋯H—O hydrogen bonds involving the solvent methanol mol­ecules (Table 1 ▸), forming a three-dimensional network.

## Synthesis and crystallization   

### 1-Phenyl-3-methyl-4-[(quinolin-3-imino)­meth­yl]-1*H*-pyrazol-5(4*H*)-one   

A solution containing 1.44 g (0.01 mol) of 3-amino­quinoline in 10 ml of toluene was added to a solution of 2.02 g (0.01 mol) of 1-phenyl-3-methyl-4-formyl­pyrazol-5-one in 20 ml of toluene. The mixture was refluxed for 3 h with a Dean–Stark trap until water stripping was completed. Subsequently, two-thirds of the total volume was distilled off on a rotary evaporator. The precipitate which formed was filtered off and recrystallized from ethanol to give light-yellow crystals (m.p. 473–474 K; yield 84%). FT–IR in KBr (ν_max_, cm^−1^): 1664 ν(C=O), 1627 δ(NH). ^1^H NMR (600 MHz, DMSO-*d*_6_, 300 K): δ 2.31 (3H, *s*, CH_3_), 7.08–8.03 (9H, *m*, C_Ar-H_), 8.52 (1H, *s*, H^4^_quin_), 8.89 (1H, *d*, *J* ^3^ = 2.7 Hz, C*H*—NH), 11.46 (1H, *br d*, *J* ^3^ = 2.7 Hz, CH—N*H*). UV–vis spectrum (nm): 232, 254, 358. PL spectrum (nm): λ_PL_ = 454, 534, λ_ex_ = 450 nm. Quantum yield of PL φ = 0.002. Analysis calculated for C_20_H_16_N_4_O: C 73.15, H 4.91, N 17.06%; found: C 73.25, H 5.10, N 17.18%.

### Bis{1-phenyl-3-methyl-4-[(quinolin-3-yl)imino­meth­yl]-1*H*-pyrazol-5-olato}zinc, (I)   

**Figure 5:**
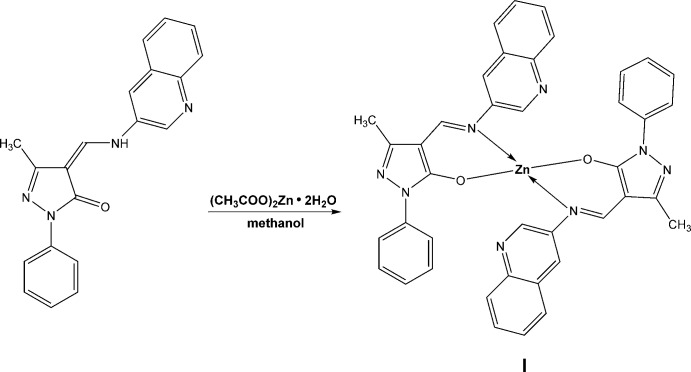
Synthesis scheme to obtain zinc complex **I**.

A hot solution of 0.22 g of zinc acetate dihydrate (1 mmol) in 20 ml of methanol was added to hot solutions of **I** (0.66 g, 2 mmol) in 20 ml of the same solvent (Fig. 5 ▸). The reaction mixture was refluxed for 2 h. The precipitates of complexes were filtered off, washed three times with 10 ml of hot methanol and dried *in vacuo*. All products were crystallized from a chloro­form–methanol (1:2 *v*/*v*) mixture and dried at 423 K, resulting in a yellow crystalline powder (m.p. 483–484 K, yield 45%). FT–IR (ν_max_, cm^−1^): 1608 ν(C=N). ^1^H NMR (600 MHz, DMSO-*d*_6_, 300 K): δ 2.25 (6H, *s*, CH_3_), 6.99–8.92 (22H, *m*, CH), 8.46 (2H, *s*, HC=N). UV–vis (nm): 360, 340, 304. PL (nm): λ_PL_ = 478, λ_ex_ = 450 nm. Analysis calculated for C_40_H_30_N_8_O_2_Zn: C 66.72, H 4.20, N 15.56%; found: C 66.78, H 4.25, N 15.64, Zn 9.11%.

## Refinement   

Crystal data, data collection and structure refinement details are summarized in Table 2 ▸.

The X-ray diffraction study was carried out on the ‘Belok’ beamline of the National Research Center ‘Kurchatov Institute’ (Moscow, Russian Federation) using a Rayonix SX165 CCD detector. A total of 360 images were collected using an oscillation range of 1.0° (φ scan mode, two different crystal orientations) and corrected for absorption using the *Scala* program (Evans, 2006 ▸). The data were indexed, integrated and scaled using the utility *iMOSFLM* in the CCP4 program (Battye *et al.*, 2011 ▸).

The data completeness of 97.8% is caused by the low (triclinic) crystal symmetry. It is very difficult to get a high data completeness for this symmetry using the φ scan mode only (‘Belok’ beamline limitation), even though we have run two different crystal orientations.

A rather large number of reflections have been omitted from refinement due to the following reasons. (i) In order to achieve better *I*/σ statistics for high-angle reflections, we selected an exposure time so as to admit a minor fraction of intensity overloads in the low-angle part of the detector. These low-angle reflections have imprecisely measured intensities and thus were excluded from the final steps of refinement. (ii) In the present set-up of the synchrotron diffractometer, the low-temperature device eclipses a small region of the 2D detector near the high-angle limit. This small shadowed region has not been masked during integration of the diffraction frames, which erroneously resulted in zero intensity of some reflections. (iii) The quality of the single crystal chosen for the diffraction experiment was not perfect. Some systematic differences between the calculated and observed intensities are probably caused by extinction and defects present in the crystal specimen.

The H atoms of the hy­droxy groups were localized from difference Fourier maps and included in a riding mode, with fixed displacement parameters [*U*_iso_(H) = 1.5*U*_eq_(O)]. All other H atoms were placed in calculated positions, with C—H = 0.95–0.98 Å, and refined in a riding mode, with fixed isotropic displacement parameters [*U*_iso_(H) = 1.5*U*_eq_(C) for the CH_3_ groups and 1.2*U*_eq_(C) for the other groups]. Disorder over two sets of sites was observed for one methanol solvent mol­ecule (atoms O7–C83). In the last cycles of refinement, the occupancy ratio was fixed at 0.75:0.25 and each of the non-H atoms was modelled with a common displacement ellipsoid.

## Supplementary Material

Crystal structure: contains datablock(s) global, I. DOI: 10.1107/S2056989017010441/wm5401sup1.cif

Structure factors: contains datablock(s) I. DOI: 10.1107/S2056989017010441/wm5401Isup2.hkl

CCDC reference: 1562057

Additional supporting information:  crystallographic information; 3D view; checkCIF report

## Figures and Tables

**Table 1:** Hydrogen-bond geometry (Å, °)

*D*—H⋯*A*	*D*—H	H⋯*A*	*D*⋯*A*	*D*—H⋯*A*
C5—H5*A*⋯O5^i^	0.98	2.43	3.312 (4)	149
C6—H6⋯O2	0.95	2.25	3.148 (4)	157
C23—H23⋯O6^ii^	0.95	2.31	3.260 (3)	179
C46—H46⋯N13	0.95	2.56	3.368 (3)	144
C66—H66⋯O3	0.95	2.46	3.344 (4)	155
C68—H68⋯O9^iii^	0.95	2.40	3.334 (4)	170
O5—H5*O*⋯N3^iv^	0.91	2.10	2.977 (4)	164
O6—H6*O*⋯N4	0.91	1.92	2.830 (3)	175
O7—H7*O*⋯N8	0.91	2.10	2.993 (4)	168
O7′—H7*OA*⋯N8	0.90	1.89	2.794 (10)	179
O8—H8*O*⋯N12^v^	0.91	1.91	2.816 (3)	173
O9—H9*O*⋯O8	0.90	1.73	2.622 (3)	170
C85—H85*C*⋯O9^vi^	0.98	2.46	3.340 (4)	150

Symmetry codes: (i) 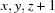; (ii) 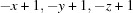; (iii) 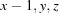; (iv) 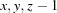; (v) 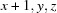; (vi) 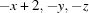.

**Table 2:** Experimental details

Crystal data	
Chemical formula	[Zn(C_20_H_15_N_4_O)_2_]·2.5CH_4_O
*M*_r_	800.20
Crystal system, space group	Triclinic, *P* 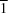
Temperature (K)	100
*a*, *b*, *c* (Å)	15.569 (3), 16.994 (3), 17.035 (3)
α, β, γ (°)	111.56 (3), 114.71 (3), 96.30 (3)
*V* (Å^3^)	3618.1 (16)
*Z*	4
Radiation type	Synchrotron, λ = 0.96990 Å
μ (mm^−1^)	1.68
Crystal size (mm)	0.20 × 0.12 × 0.07

Data collection	
Diffractometer	Rayonix SX165 CCD
Absorption correction	Multi-scan (*SCALA*; Evans, 2006 ▸)
*T*_min_, *T*_max_	0.730, 0.880
No. of measured, independent and observed [*I* > 2σ(*I*)] reflections	48608, 15616, 12285
*R*_int_	0.055
(sin θ/λ)_max_ (Å^−1^)	0.682

Refinement	
*R*[*F* ^2^ > 2σ(*F* ^2^)], *wR*(*F* ^2^), *S*	0.052, 0.152, 1.10
No. of reflections	15616
No. of parameters	1020
No. of restraints	9
H-atom treatment	H-atom parameters constrained
Δρ_max_, Δρ_min_ (e Å^−3^)	1.03, −1.00

Computer programs: *Marccd* (Doyle, 2011 ▸), *iMosflm* (Battye *et al.*, 2011 ▸), *SHELXT* (Sheldrick, 2015*a* ▸), *SHELXL2014* (Sheldrick, 2015*b* ▸) and *SHELXTL* (Sheldrick, 2008 ▸).

## supplementary crystallographic information

### Crystal data

**Table:**

[Zn(C_20_H_15_N_4_O)_2_]·2.5CH_4_O	*Z* = 4
*M**_r_* = 800.20	*F*(000) = 1668
Triclinic, *P*1	*D*_x_ = 1.469 Mg m^−^^3^
*a* = 15.569 (3) Å	Synchrotron radiation, λ = 0.96990 Å
*b* = 16.994 (3) Å	Cell parameters from 600 reflections
*c* = 17.035 (3) Å	θ = 3.3–33.0°
α = 111.56 (3)°	µ = 1.68 mm^−^^1^
β = 114.71 (3)°	*T* = 100 K
γ = 96.30 (3)°	Prism, yellow
*V* = 3618.1 (16) Å^3^	0.20 × 0.12 × 0.07 mm

### Data collection

**Table:**

Rayonix SX165 CCD diffractometer	12285 reflections with *I* > 2σ(*I*)
/f scan	*R*_int_ = 0.055
Absorption correction: multi-scan (*SCALA*; Evans, 2006)	θ_max_ = 41.5°, θ_min_ = 3.3°
*T*_min_ = 0.730, *T*_max_ = 0.880	*h* = −19→21
48608 measured reflections	*k* = −21→21
15616 independent reflections	*l* = −20→20

### Refinement

**Table:**

Refinement on *F*^2^	Secondary atom site location: difference Fourier map
Least-squares matrix: full	Hydrogen site location: mixed
*R*[*F*^2^ > 2σ(*F*^2^)] = 0.052	H-atom parameters constrained
*wR*(*F*^2^) = 0.152	*w* = 1/[σ^2^(*F*_o_^2^) + (0.0757*P*)^2^] where *P* = (*F*_o_^2^ + 2*F*_c_^2^)/3
*S* = 1.10	(Δ/σ)_max_ = 0.001
15616 reflections	Δρ_max_ = 1.03 e Å^−^^3^
1020 parameters	Δρ_min_ = −1.00 e Å^−^^3^
9 restraints	Extinction correction: SHELXL2014 (Sheldrick, 2015b), Fc^*^=kFc[1+0.001xFc^2^λ^3^/sin(2θ)]^-1/4^
Primary atom site location: difference Fourier map	Extinction coefficient: 0.0043 (4)

### Special details

**Table:**

Geometry. All esds (except the esd in the dihedral angle between two l.s. planes) are estimated using the full covariance matrix. The cell esds are taken into account individually in the estimation of esds in distances, angles and torsion angles; correlations between esds in cell parameters are only used when they are defined by crystal symmetry. An approximate (isotropic) treatment of cell esds is used for estimating esds involving l.s. planes.

### Fractional atomic coordinates and isotropic or equivalent isotropic displacement parameters (Å^2^)

**Table:**

	*x*	*y*	*z*	*U*_iso_*/*U*_eq_	Occ. (<1)
Zn1	0.37780 (2)	0.39436 (2)	0.67054 (2)	0.02024 (11)	
O1	0.29175 (13)	0.38716 (12)	0.72004 (13)	0.0238 (4)	
O2	0.35484 (12)	0.29575 (11)	0.54978 (13)	0.0209 (4)	
N1	0.49660 (15)	0.40526 (14)	0.78587 (16)	0.0205 (5)	
N2	0.26374 (15)	0.36528 (14)	0.83484 (16)	0.0214 (5)	
N3	0.31383 (16)	0.36130 (14)	0.92341 (16)	0.0218 (5)	
N4	0.66016 (16)	0.38308 (14)	0.69054 (17)	0.0243 (5)	
N5	0.37862 (15)	0.49341 (13)	0.63202 (16)	0.0206 (5)	
N6	0.34901 (15)	0.25383 (13)	0.39951 (16)	0.0207 (5)	
N7	0.34356 (16)	0.29006 (14)	0.33570 (16)	0.0231 (5)	
N8	0.36736 (15)	0.67970 (14)	0.82180 (16)	0.0232 (5)	
C1	0.32160 (18)	0.38125 (16)	0.80065 (19)	0.0192 (5)	
C2	0.41154 (18)	0.38661 (16)	0.86662 (19)	0.0194 (5)	
C3	0.49342 (18)	0.39740 (15)	0.85906 (19)	0.0207 (5)	
H3	0.5531	0.3996	0.9094	0.025*	
C4	0.40116 (19)	0.37385 (16)	0.94125 (19)	0.0215 (5)	
C5	0.4747 (2)	0.37338 (18)	1.0299 (2)	0.0272 (6)	
H5A	0.4439	0.3699	1.0688	0.041*	
H5B	0.5311	0.4282	1.0679	0.041*	
H5C	0.4981	0.3218	1.0123	0.041*	
C6	0.58466 (19)	0.37789 (17)	0.6995 (2)	0.0229 (6)	
H6	0.5220	0.3447	0.6420	0.028*	
C7	0.58503 (18)	0.41789 (16)	0.7891 (2)	0.0208 (5)	
C8	0.66931 (19)	0.46668 (17)	0.8722 (2)	0.0242 (6)	
H8	0.6705	0.4938	0.9325	0.029*	
C9	0.75239 (19)	0.47558 (16)	0.8663 (2)	0.0253 (6)	
C10	0.8428 (2)	0.52813 (18)	0.9490 (2)	0.0320 (7)	
H10	0.8479	0.5562	1.0110	0.038*	
C11	0.9204 (2)	0.53763 (19)	0.9387 (3)	0.0361 (7)	
H11	0.9835	0.5743	0.9942	0.043*	
C12	0.9134 (2)	0.4946 (2)	0.8465 (3)	0.0356 (7)	
H12	0.9724	0.5032	0.8423	0.043*	
C13	0.8272 (2)	0.44248 (19)	0.7652 (2)	0.0296 (6)	
H13	0.8239	0.4137	0.7040	0.036*	
C14	0.74590 (18)	0.43292 (16)	0.7742 (2)	0.0229 (6)	
C15	0.16479 (18)	0.34483 (16)	0.7905 (2)	0.0219 (5)	
C16	0.1179 (2)	0.30312 (17)	0.8231 (2)	0.0267 (6)	
H16	0.1545	0.2903	0.8750	0.032*	
C17	0.0215 (2)	0.28156 (18)	0.7807 (2)	0.0302 (6)	
H17	−0.0139	0.2528	0.8016	0.036*	
C18	−0.0290 (2)	0.30057 (19)	0.7053 (2)	0.0326 (7)	
H18	−0.0996	0.2835	0.6744	0.039*	
C19	0.0175 (2)	0.3428 (2)	0.6730 (2)	0.0368 (7)	
H19	−0.0196	0.3552	0.6208	0.044*	
C20	0.1150 (2)	0.3659 (2)	0.7157 (2)	0.0325 (7)	
H20	0.1504	0.3962	0.6960	0.039*	
C21	0.35394 (17)	0.31461 (16)	0.48261 (19)	0.0199 (5)	
C22	0.35634 (18)	0.39531 (16)	0.4747 (2)	0.0208 (5)	
C23	0.36918 (18)	0.47910 (16)	0.5466 (2)	0.0208 (5)	
H23	0.3711	0.5281	0.5324	0.025*	
C24	0.34803 (19)	0.37380 (17)	0.3816 (2)	0.0223 (5)	
C25	0.3440 (2)	0.43470 (18)	0.3360 (2)	0.0293 (6)	
H25A	0.4054	0.4852	0.3772	0.044*	
H25B	0.2869	0.4565	0.3294	0.044*	
H25C	0.3371	0.4022	0.2717	0.044*	
C26	0.34980 (18)	0.60058 (17)	0.75209 (19)	0.0221 (5)	
H26	0.2977	0.5524	0.7356	0.026*	
C27	0.40126 (18)	0.58290 (16)	0.70177 (19)	0.0198 (5)	
C28	0.47348 (19)	0.65111 (16)	0.7258 (2)	0.0232 (6)	
H28	0.5102	0.6431	0.6924	0.028*	
C29	0.49628 (19)	0.73673 (17)	0.8020 (2)	0.0243 (6)	
C30	0.5750 (2)	0.80897 (18)	0.8366 (2)	0.0309 (6)	
H30	0.6143	0.8038	0.8059	0.037*	
C31	0.5984 (2)	0.88879 (19)	0.9149 (2)	0.0339 (7)	
H31	0.6544	0.9367	0.9376	0.041*	
C32	0.5434 (2)	0.90042 (18)	0.9601 (2)	0.0300 (6)	
H32	0.5604	0.9556	1.0141	0.036*	
C33	0.4658 (2)	0.83314 (18)	0.9272 (2)	0.0266 (6)	
H33	0.4254	0.8410	0.9568	0.032*	
C34	0.44091 (18)	0.74835 (17)	0.84777 (19)	0.0219 (5)	
C35	0.35835 (18)	0.16739 (16)	0.3774 (2)	0.0236 (6)	
C36	0.3223 (2)	0.10542 (17)	0.2805 (2)	0.0287 (6)	
H36	0.2895	0.1205	0.2295	0.034*	
C37	0.3349 (2)	0.02128 (19)	0.2594 (3)	0.0363 (7)	
H37	0.3116	−0.0207	0.1940	0.044*	
C38	0.3812 (2)	−0.00050 (19)	0.3338 (3)	0.0392 (8)	
H38	0.3894	−0.0578	0.3194	0.047*	
C39	0.4160 (2)	0.06077 (19)	0.4293 (3)	0.0366 (7)	
H39	0.4474	0.0446	0.4797	0.044*	
C40	0.4059 (2)	0.14537 (18)	0.4529 (2)	0.0297 (6)	
H40	0.4306	0.1872	0.5187	0.036*	
Zn2	0.14334 (2)	0.19055 (2)	0.49603 (2)	0.02038 (11)	
O3	0.25041 (12)	0.18339 (12)	0.59848 (13)	0.0232 (4)	
O4	0.14119 (13)	0.30882 (11)	0.50551 (13)	0.0220 (4)	
N9	0.03944 (15)	0.14139 (13)	0.51630 (16)	0.0200 (4)	
N10	0.30601 (15)	0.14766 (13)	0.72752 (16)	0.0199 (4)	
N11	0.27194 (15)	0.11814 (14)	0.77823 (16)	0.0216 (5)	
N12	−0.16119 (16)	0.13409 (14)	0.30661 (17)	0.0251 (5)	
N13	0.13225 (15)	0.12821 (14)	0.36285 (16)	0.0209 (5)	
N14	0.12460 (15)	0.39837 (14)	0.42710 (16)	0.0223 (5)	
N15	0.11355 (16)	0.39342 (15)	0.33822 (17)	0.0247 (5)	
N16	0.20286 (16)	−0.07021 (15)	0.36424 (18)	0.0273 (5)	
C41	0.23629 (18)	0.15527 (15)	0.65373 (19)	0.0202 (5)	
C42	0.15239 (18)	0.12730 (16)	0.65418 (19)	0.0193 (5)	
C43	0.06198 (18)	0.12112 (16)	0.58986 (19)	0.0213 (5)	
H43	0.0080	0.1001	0.5972	0.026*	
C44	0.18157 (18)	0.10603 (16)	0.73460 (19)	0.0202 (5)	
C45	0.1212 (2)	0.07455 (18)	0.7689 (2)	0.0258 (6)	
H45A	0.1618	0.0589	0.8194	0.039*	
H45B	0.0654	0.0219	0.7147	0.039*	
H45C	0.0957	0.1215	0.7960	0.039*	
C46	−0.07672 (19)	0.14521 (17)	0.3731 (2)	0.0237 (6)	
H46	−0.0211	0.1649	0.3674	0.028*	
C47	−0.05610 (18)	0.13132 (16)	0.45597 (19)	0.0202 (5)	
C48	−0.12988 (18)	0.10836 (16)	0.4694 (2)	0.0221 (5)	
H48	−0.1192	0.1016	0.5254	0.027*	
C49	−0.22218 (18)	0.09481 (16)	0.39962 (19)	0.0217 (5)	
C50	−0.30175 (19)	0.06775 (17)	0.4078 (2)	0.0268 (6)	
H50	−0.2944	0.0598	0.4623	0.032*	
C51	−0.38957 (19)	0.05313 (18)	0.3365 (2)	0.0289 (6)	
H51	−0.4469	0.0330	0.3396	0.035*	
C52	−0.4029 (2)	0.06606 (18)	0.2548 (2)	0.0301 (6)	
H52	−0.4683	0.0544	0.2054	0.036*	
C53	−0.32737 (19)	0.09351 (18)	0.2461 (2)	0.0269 (6)	
H53	−0.3362	0.1030	0.1920	0.032*	
C54	−0.23574 (19)	0.10789 (16)	0.3178 (2)	0.0230 (6)	
C55	0.40251 (16)	0.16565 (15)	0.75695 (15)	0.0203 (5)	
C56	0.46266 (15)	0.17187 (18)	0.84735 (17)	0.0276 (6)	
H56	0.4358	0.1625	0.8852	0.033*	
C57	0.56265 (16)	0.1922 (2)	0.8796 (2)	0.0332 (7)	
H57	0.6084	0.1990	0.9418	0.040*	
C58	0.59480 (18)	0.20255 (18)	0.81881 (16)	0.0294 (6)	
H58	0.6642	0.2162	0.8411	0.035*	
C59	0.53357 (15)	0.19451 (16)	0.72805 (17)	0.0271 (6)	
H59	0.5603	0.2021	0.6894	0.032*	
C60	0.43374 (15)	0.17540 (17)	0.69502 (18)	0.0239 (6)	
H60	0.3883	0.1692	0.6331	0.029*	
C61	0.12851 (17)	0.31969 (16)	0.43077 (19)	0.0210 (5)	
C62	0.11890 (18)	0.26028 (16)	0.34075 (19)	0.0209 (5)	
C63	0.12335 (18)	0.17260 (17)	0.3114 (2)	0.0224 (5)	
H63	0.1197	0.1429	0.2502	0.027*	
C64	0.11014 (19)	0.31161 (17)	0.2881 (2)	0.0245 (6)	
C65	0.0995 (2)	0.28071 (19)	0.1886 (2)	0.0313 (6)	
H65A	0.0934	0.3288	0.1700	0.047*	
H65B	0.0399	0.2288	0.1414	0.047*	
H65C	0.1583	0.2644	0.1897	0.047*	
C66	0.18713 (19)	0.00784 (17)	0.3892 (2)	0.0245 (6)	
H66	0.2111	0.0450	0.4562	0.029*	
C67	0.13683 (18)	0.03949 (16)	0.3224 (2)	0.0215 (5)	
C68	0.09663 (19)	−0.01641 (17)	0.2251 (2)	0.0243 (6)	
H68	0.0575	0.0010	0.1782	0.029*	
C69	0.11342 (18)	−0.10023 (17)	0.1943 (2)	0.0233 (6)	
C70	0.0797 (2)	−0.15916 (18)	0.0951 (2)	0.0297 (6)	
H70	0.0413	−0.1440	0.0458	0.036*	
C71	0.1020 (2)	−0.23704 (19)	0.0701 (2)	0.0313 (6)	
H71	0.0797	−0.2756	0.0039	0.038*	
C72	0.1582 (2)	−0.25962 (17)	0.1430 (2)	0.0299 (7)	
H72	0.1737	−0.3138	0.1256	0.036*	
C73	0.19092 (19)	−0.20465 (17)	0.2388 (2)	0.0271 (6)	
H73	0.2287	−0.2213	0.2869	0.032*	
C74	0.16926 (18)	−0.12328 (17)	0.2671 (2)	0.0239 (6)	
C75	0.13075 (18)	0.47956 (17)	0.4985 (2)	0.0238 (6)	
C76	0.16578 (19)	0.55997 (17)	0.5011 (2)	0.0273 (6)	
H76	0.1882	0.5610	0.4574	0.033*	
C77	0.1678 (2)	0.63934 (18)	0.5686 (2)	0.0327 (7)	
H77	0.1908	0.6940	0.5697	0.039*	
C78	0.1373 (2)	0.63923 (19)	0.6327 (2)	0.0323 (7)	
H78	0.1396	0.6934	0.6787	0.039*	
C79	0.1029 (2)	0.55904 (18)	0.6298 (2)	0.0283 (6)	
H79	0.0813	0.5585	0.6741	0.034*	
C80	0.09951 (18)	0.47912 (18)	0.5630 (2)	0.0249 (6)	
H80	0.0758	0.4246	0.5620	0.030*	
O5	0.2941 (2)	0.33191 (18)	0.0777 (2)	0.0621 (7)	
H5O	0.2883	0.3431	0.0280	0.093*	
C81	0.2308 (3)	0.2495 (3)	0.0424 (3)	0.0624 (11)	
H81A	0.2412	0.2050	−0.0061	0.094*	
H81B	0.1618	0.2499	0.0121	0.094*	
H81C	0.2439	0.2346	0.0958	0.094*	
O6	0.6250 (2)	0.35347 (13)	0.50351 (17)	0.0479 (6)	
H6O	0.6406	0.3638	0.5651	0.072*	
C82	0.6201 (3)	0.26479 (19)	0.4497 (3)	0.0420 (8)	
H82A	0.6318	0.2609	0.3963	0.063*	
H82B	0.5541	0.2243	0.4231	0.063*	
H82C	0.6709	0.2480	0.4925	0.063*	
O7	0.3036 (2)	0.5971 (2)	0.9244 (3)	0.0512 (7)	0.75
H7O	0.3224	0.6295	0.8988	0.077*	0.75
C83	0.2137 (3)	0.6052 (4)	0.9191 (4)	0.0512 (7)	0.75
H83A	0.1836	0.5579	0.9282	0.077*	0.75
H83B	0.1687	0.6000	0.8552	0.077*	0.75
H83C	0.2255	0.6633	0.9700	0.077*	0.75
O7'	0.2416 (7)	0.6655 (6)	0.8977 (7)	0.0512 (7)	0.25
H7OA	0.2827	0.6710	0.8741	0.077*	0.25
C83'	0.2430 (14)	0.5920 (9)	0.9165 (14)	0.0512 (7)	0.25
H83D	0.2104	0.5936	0.9552	0.077*	0.25
H83E	0.3120	0.5938	0.9526	0.077*	0.25
H83F	0.2075	0.5370	0.8550	0.077*	0.25
O8	0.8195 (2)	0.1154 (2)	0.1282 (2)	0.0642 (8)	
H8O	0.8273	0.1264	0.1876	0.096*	
C84	0.8099 (4)	0.1902 (3)	0.1137 (4)	0.0820 (16)	
H84A	0.7969	0.1768	0.0479	0.123*	
H84B	0.8715	0.2398	0.1614	0.123*	
H84C	0.7544	0.2065	0.1215	0.123*	
O9	0.94341 (16)	0.05123 (15)	0.07636 (16)	0.0444 (6)	
H9O	0.9059	0.0749	0.1013	0.067*	
C85	0.8816 (3)	−0.0250 (2)	−0.0130 (3)	0.0488 (9)	
H85A	0.8393	−0.0075	−0.0609	0.073*	
H85B	0.8397	−0.0651	−0.0047	0.073*	
H85C	0.9227	−0.0556	−0.0357	0.073*	

### Atomic displacement parameters (Å^2^)

**Table:**

	*U*^11^	*U*^22^	*U*^33^	*U*^12^	*U*^13^	*U*^23^
Zn1	0.01949 (16)	0.02475 (17)	0.0224 (2)	0.00955 (13)	0.01238 (15)	0.01354 (15)
O1	0.0200 (9)	0.0322 (10)	0.0250 (11)	0.0102 (7)	0.0123 (8)	0.0169 (9)
O2	0.0211 (9)	0.0222 (8)	0.0222 (10)	0.0074 (7)	0.0113 (8)	0.0122 (8)
N1	0.0168 (10)	0.0236 (10)	0.0230 (13)	0.0090 (8)	0.0105 (9)	0.0110 (10)
N2	0.0171 (10)	0.0266 (11)	0.0236 (13)	0.0076 (8)	0.0102 (9)	0.0143 (10)
N3	0.0231 (11)	0.0263 (11)	0.0208 (12)	0.0092 (9)	0.0126 (10)	0.0129 (10)
N4	0.0206 (11)	0.0297 (11)	0.0298 (14)	0.0109 (9)	0.0149 (10)	0.0167 (11)
N5	0.0184 (10)	0.0235 (10)	0.0231 (13)	0.0090 (8)	0.0116 (10)	0.0116 (10)
N6	0.0198 (10)	0.0222 (10)	0.0233 (13)	0.0087 (8)	0.0120 (10)	0.0114 (10)
N7	0.0229 (11)	0.0278 (11)	0.0232 (13)	0.0095 (9)	0.0130 (10)	0.0140 (10)
N8	0.0180 (10)	0.0300 (11)	0.0212 (13)	0.0102 (9)	0.0096 (10)	0.0108 (10)
C1	0.0216 (12)	0.0201 (11)	0.0207 (14)	0.0077 (10)	0.0139 (11)	0.0099 (11)
C2	0.0203 (12)	0.0213 (12)	0.0193 (14)	0.0090 (10)	0.0108 (11)	0.0101 (11)
C3	0.0198 (12)	0.0201 (12)	0.0221 (15)	0.0086 (10)	0.0101 (11)	0.0093 (11)
C4	0.0235 (12)	0.0229 (12)	0.0209 (15)	0.0099 (10)	0.0117 (11)	0.0111 (11)
C5	0.0288 (14)	0.0333 (14)	0.0261 (16)	0.0140 (12)	0.0146 (13)	0.0175 (13)
C6	0.0192 (12)	0.0264 (13)	0.0278 (16)	0.0092 (10)	0.0133 (12)	0.0145 (12)
C7	0.0182 (12)	0.0233 (12)	0.0283 (16)	0.0112 (10)	0.0133 (12)	0.0158 (12)
C8	0.0230 (13)	0.0257 (13)	0.0197 (15)	0.0072 (11)	0.0094 (12)	0.0078 (12)
C9	0.0201 (12)	0.0225 (12)	0.0305 (17)	0.0077 (10)	0.0104 (12)	0.0117 (12)
C10	0.0208 (13)	0.0301 (14)	0.0351 (18)	0.0066 (11)	0.0099 (13)	0.0103 (13)
C11	0.0202 (13)	0.0312 (15)	0.051 (2)	0.0069 (12)	0.0127 (14)	0.0190 (15)
C12	0.0189 (13)	0.0429 (17)	0.053 (2)	0.0122 (12)	0.0180 (14)	0.0290 (16)
C13	0.0243 (13)	0.0368 (15)	0.0407 (18)	0.0158 (12)	0.0204 (14)	0.0238 (14)
C14	0.0196 (12)	0.0251 (12)	0.0291 (16)	0.0104 (10)	0.0127 (12)	0.0157 (12)
C15	0.0169 (12)	0.0235 (12)	0.0249 (15)	0.0076 (10)	0.0111 (11)	0.0095 (11)
C16	0.0249 (13)	0.0268 (13)	0.0327 (17)	0.0076 (11)	0.0169 (13)	0.0149 (13)
C17	0.0238 (13)	0.0313 (14)	0.0359 (18)	0.0052 (11)	0.0170 (13)	0.0141 (14)
C18	0.0214 (13)	0.0364 (15)	0.0381 (19)	0.0092 (12)	0.0167 (13)	0.0129 (14)
C19	0.0232 (14)	0.0553 (19)	0.042 (2)	0.0165 (13)	0.0174 (14)	0.0300 (17)
C20	0.0206 (13)	0.0479 (17)	0.0380 (19)	0.0122 (12)	0.0165 (13)	0.0257 (15)
C21	0.0131 (11)	0.0232 (12)	0.0233 (15)	0.0062 (9)	0.0078 (11)	0.0119 (11)
C22	0.0205 (12)	0.0225 (12)	0.0237 (15)	0.0087 (10)	0.0125 (11)	0.0125 (11)
C23	0.0188 (12)	0.0217 (12)	0.0268 (16)	0.0080 (10)	0.0120 (11)	0.0143 (11)
C24	0.0211 (12)	0.0252 (12)	0.0233 (15)	0.0089 (10)	0.0126 (12)	0.0117 (12)
C25	0.0379 (16)	0.0318 (14)	0.0298 (17)	0.0156 (12)	0.0215 (14)	0.0184 (13)
C26	0.0176 (12)	0.0287 (13)	0.0200 (15)	0.0094 (10)	0.0091 (11)	0.0108 (12)
C27	0.0185 (11)	0.0228 (12)	0.0203 (14)	0.0110 (10)	0.0088 (11)	0.0118 (11)
C28	0.0227 (12)	0.0261 (13)	0.0257 (15)	0.0097 (10)	0.0141 (12)	0.0136 (12)
C29	0.0226 (13)	0.0254 (13)	0.0264 (16)	0.0088 (10)	0.0117 (12)	0.0136 (12)
C30	0.0270 (14)	0.0298 (14)	0.0377 (18)	0.0082 (12)	0.0172 (14)	0.0158 (14)
C31	0.0307 (15)	0.0266 (14)	0.0384 (19)	0.0069 (12)	0.0149 (14)	0.0120 (14)
C32	0.0312 (15)	0.0244 (13)	0.0243 (16)	0.0100 (12)	0.0088 (13)	0.0067 (12)
C33	0.0276 (14)	0.0326 (14)	0.0213 (15)	0.0165 (12)	0.0116 (12)	0.0126 (12)
C34	0.0196 (12)	0.0274 (13)	0.0180 (14)	0.0114 (10)	0.0061 (11)	0.0124 (11)
C35	0.0178 (12)	0.0210 (12)	0.0335 (17)	0.0065 (10)	0.0153 (12)	0.0108 (12)
C36	0.0249 (13)	0.0252 (13)	0.0361 (18)	0.0060 (11)	0.0198 (13)	0.0094 (13)
C37	0.0275 (15)	0.0264 (14)	0.044 (2)	0.0028 (12)	0.0224 (15)	0.0020 (14)
C38	0.0328 (16)	0.0260 (14)	0.072 (3)	0.0150 (13)	0.0360 (18)	0.0219 (17)
C39	0.0353 (16)	0.0323 (15)	0.057 (2)	0.0195 (13)	0.0282 (16)	0.0259 (16)
C40	0.0268 (14)	0.0285 (14)	0.0421 (19)	0.0142 (11)	0.0209 (14)	0.0182 (14)
Zn2	0.01867 (16)	0.02417 (17)	0.0226 (2)	0.00916 (12)	0.01069 (14)	0.01370 (15)
O3	0.0187 (9)	0.0312 (10)	0.0266 (11)	0.0098 (7)	0.0114 (8)	0.0192 (9)
O4	0.0226 (9)	0.0248 (9)	0.0225 (10)	0.0102 (7)	0.0112 (8)	0.0136 (8)
N9	0.0171 (10)	0.0232 (10)	0.0222 (12)	0.0097 (8)	0.0107 (9)	0.0107 (10)
N10	0.0167 (10)	0.0232 (10)	0.0222 (12)	0.0078 (8)	0.0094 (9)	0.0126 (10)
N11	0.0200 (10)	0.0242 (11)	0.0241 (13)	0.0079 (9)	0.0115 (10)	0.0135 (10)
N12	0.0214 (11)	0.0266 (11)	0.0274 (14)	0.0101 (9)	0.0114 (10)	0.0124 (10)
N13	0.0159 (10)	0.0235 (10)	0.0246 (13)	0.0076 (8)	0.0092 (9)	0.0128 (10)
N14	0.0206 (10)	0.0244 (11)	0.0249 (13)	0.0101 (9)	0.0107 (10)	0.0138 (10)
N15	0.0266 (11)	0.0311 (12)	0.0220 (13)	0.0117 (10)	0.0125 (10)	0.0161 (10)
N16	0.0248 (11)	0.0294 (12)	0.0309 (15)	0.0114 (10)	0.0141 (11)	0.0159 (11)
C41	0.0215 (12)	0.0182 (11)	0.0198 (14)	0.0077 (10)	0.0092 (11)	0.0084 (11)
C42	0.0182 (11)	0.0193 (11)	0.0210 (14)	0.0063 (9)	0.0096 (11)	0.0098 (11)
C43	0.0214 (12)	0.0204 (12)	0.0252 (15)	0.0091 (10)	0.0135 (12)	0.0103 (11)
C44	0.0208 (12)	0.0190 (11)	0.0211 (14)	0.0076 (10)	0.0098 (11)	0.0099 (11)
C45	0.0248 (13)	0.0312 (14)	0.0276 (16)	0.0118 (11)	0.0142 (12)	0.0171 (13)
C46	0.0199 (12)	0.0290 (13)	0.0240 (16)	0.0104 (10)	0.0108 (12)	0.0134 (12)
C47	0.0172 (11)	0.0200 (12)	0.0215 (15)	0.0080 (10)	0.0081 (11)	0.0088 (11)
C48	0.0225 (12)	0.0249 (12)	0.0214 (15)	0.0096 (10)	0.0110 (11)	0.0124 (11)
C49	0.0185 (12)	0.0192 (11)	0.0251 (15)	0.0079 (10)	0.0097 (11)	0.0087 (11)
C50	0.0211 (13)	0.0281 (13)	0.0309 (17)	0.0081 (11)	0.0112 (12)	0.0151 (13)
C51	0.0189 (13)	0.0307 (14)	0.0350 (18)	0.0083 (11)	0.0122 (13)	0.0141 (13)
C52	0.0212 (13)	0.0291 (14)	0.0327 (18)	0.0111 (11)	0.0084 (13)	0.0120 (13)
C53	0.0222 (13)	0.0308 (14)	0.0232 (16)	0.0121 (11)	0.0069 (12)	0.0119 (12)
C54	0.0223 (12)	0.0198 (12)	0.0244 (15)	0.0089 (10)	0.0114 (12)	0.0072 (11)
C55	0.0173 (11)	0.0172 (11)	0.0245 (15)	0.0059 (9)	0.0090 (11)	0.0090 (11)
C56	0.0214 (13)	0.0324 (14)	0.0249 (16)	0.0077 (11)	0.0094 (12)	0.0117 (13)
C57	0.0225 (14)	0.0435 (17)	0.0304 (18)	0.0116 (12)	0.0117 (13)	0.0151 (14)
C58	0.0180 (12)	0.0324 (14)	0.0330 (17)	0.0085 (11)	0.0094 (12)	0.0138 (13)
C59	0.0257 (13)	0.0245 (13)	0.0385 (18)	0.0120 (11)	0.0197 (13)	0.0160 (13)
C60	0.0222 (13)	0.0268 (13)	0.0287 (16)	0.0114 (11)	0.0131 (12)	0.0167 (12)
C61	0.0134 (11)	0.0247 (12)	0.0244 (15)	0.0075 (10)	0.0064 (11)	0.0138 (12)
C62	0.0191 (12)	0.0242 (12)	0.0204 (14)	0.0082 (10)	0.0087 (11)	0.0122 (11)
C63	0.0156 (11)	0.0241 (12)	0.0245 (15)	0.0045 (10)	0.0097 (11)	0.0090 (11)
C64	0.0214 (12)	0.0282 (13)	0.0254 (16)	0.0083 (10)	0.0106 (12)	0.0146 (12)
C65	0.0397 (16)	0.0351 (15)	0.0269 (17)	0.0149 (13)	0.0183 (14)	0.0188 (14)
C66	0.0237 (13)	0.0268 (13)	0.0241 (15)	0.0104 (11)	0.0115 (12)	0.0123 (12)
C67	0.0165 (11)	0.0219 (12)	0.0257 (15)	0.0044 (10)	0.0115 (11)	0.0098 (11)
C68	0.0213 (12)	0.0257 (13)	0.0272 (16)	0.0070 (10)	0.0125 (12)	0.0128 (12)
C69	0.0150 (11)	0.0247 (13)	0.0264 (16)	0.0023 (10)	0.0090 (11)	0.0106 (12)
C70	0.0305 (14)	0.0275 (14)	0.0266 (17)	0.0075 (12)	0.0121 (13)	0.0108 (13)
C71	0.0294 (14)	0.0286 (14)	0.0324 (18)	0.0076 (12)	0.0160 (14)	0.0102 (13)
C72	0.0234 (13)	0.0215 (13)	0.0457 (19)	0.0063 (11)	0.0214 (14)	0.0114 (13)
C73	0.0198 (12)	0.0253 (13)	0.0367 (18)	0.0066 (10)	0.0145 (12)	0.0142 (13)
C74	0.0171 (12)	0.0267 (13)	0.0296 (16)	0.0054 (10)	0.0127 (12)	0.0138 (12)
C75	0.0150 (11)	0.0261 (13)	0.0300 (16)	0.0112 (10)	0.0079 (11)	0.0149 (12)
C76	0.0219 (13)	0.0273 (13)	0.0334 (17)	0.0089 (11)	0.0123 (12)	0.0161 (13)
C77	0.0269 (14)	0.0251 (13)	0.0376 (18)	0.0093 (11)	0.0090 (14)	0.0141 (13)
C78	0.0289 (14)	0.0297 (14)	0.0288 (17)	0.0146 (12)	0.0092 (13)	0.0086 (13)
C79	0.0248 (13)	0.0344 (15)	0.0262 (16)	0.0164 (12)	0.0108 (13)	0.0145 (13)
C80	0.0192 (12)	0.0294 (13)	0.0263 (16)	0.0115 (11)	0.0081 (12)	0.0156 (12)
O5	0.0685 (18)	0.0618 (16)	0.0607 (19)	0.0147 (14)	0.0344 (15)	0.0314 (15)
C81	0.052 (2)	0.063 (2)	0.072 (3)	0.0076 (19)	0.024 (2)	0.041 (2)
O6	0.0923 (19)	0.0298 (11)	0.0359 (14)	0.0267 (12)	0.0391 (14)	0.0182 (10)
C82	0.057 (2)	0.0298 (15)	0.042 (2)	0.0132 (15)	0.0303 (18)	0.0127 (15)
O7	0.0479 (17)	0.0638 (16)	0.0593 (17)	0.0222 (14)	0.0350 (15)	0.0340 (14)
C83	0.0479 (17)	0.0638 (16)	0.0593 (17)	0.0222 (14)	0.0350 (15)	0.0340 (14)
O7'	0.0479 (17)	0.0638 (16)	0.0593 (17)	0.0222 (14)	0.0350 (15)	0.0340 (14)
C83'	0.0479 (17)	0.0638 (16)	0.0593 (17)	0.0222 (14)	0.0350 (15)	0.0340 (14)
O8	0.0617 (17)	0.109 (2)	0.0485 (17)	0.0452 (17)	0.0387 (15)	0.0440 (17)
C84	0.092 (4)	0.142 (5)	0.079 (3)	0.077 (4)	0.063 (3)	0.081 (4)
O9	0.0315 (11)	0.0557 (14)	0.0372 (14)	0.0163 (10)	0.0146 (11)	0.0148 (12)
C85	0.0362 (18)	0.050 (2)	0.049 (2)	0.0066 (15)	0.0205 (17)	0.0140 (18)

### Geometric parameters (Å, º)

**Table:**

Zn1—O1	1.8721 (18)	N13—C67	1.433 (3)
Zn1—O2	1.972 (2)	N14—C61	1.368 (3)
Zn1—N1	1.983 (2)	N14—N15	1.419 (3)
Zn1—N5	2.019 (2)	N14—C75	1.426 (3)
O1—C1	1.300 (3)	N15—C64	1.319 (3)
O2—C21	1.292 (3)	N16—C66	1.321 (3)
N1—C3	1.322 (3)	N16—C74	1.379 (4)
N1—C7	1.344 (3)	C41—C42	1.344 (3)
N2—C1	1.314 (3)	C42—C43	1.337 (4)
N2—C15	1.335 (3)	C42—C44	1.447 (4)
N2—N3	1.411 (3)	C43—H43	0.9500
N3—C4	1.237 (3)	C44—C45	1.446 (4)
N4—C6	1.245 (3)	C45—H45A	0.9800
N4—C14	1.343 (4)	C45—H45B	0.9800
N5—C23	1.323 (3)	C45—H45C	0.9800
N5—C27	1.433 (3)	C46—C47	1.426 (4)
N6—C21	1.376 (3)	C46—H46	0.9500
N6—N7	1.412 (3)	C47—C48	1.308 (3)
N6—C35	1.417 (3)	C48—C49	1.352 (4)
N7—C24	1.325 (3)	C48—H48	0.9500
N8—C26	1.329 (3)	C49—C50	1.353 (4)
N8—C34	1.331 (3)	C49—C54	1.424 (4)
C1—C2	1.346 (4)	C50—C51	1.312 (4)
C2—C3	1.334 (3)	C50—H50	0.9500
C2—C4	1.434 (4)	C51—C52	1.422 (4)
C3—H3	0.9500	C51—H51	0.9500
C4—C5	1.469 (4)	C52—C53	1.303 (4)
C5—H5A	0.9800	C52—H52	0.9500
C5—H5B	0.9800	C53—C54	1.355 (4)
C5—H5C	0.9800	C53—H53	0.9500
C6—C7	1.422 (4)	C55—C56	1.3828 (19)
C6—H6	0.9500	C55—C60	1.3845 (18)
C7—C8	1.326 (4)	C56—C57	1.3682 (19)
C8—C9	1.335 (4)	C56—H56	0.9500
C8—H8	0.9500	C57—C58	1.3795 (19)
C9—C10	1.380 (4)	C57—H57	0.9500
C9—C14	1.418 (4)	C58—C59	1.3789 (19)
C10—C11	1.292 (4)	C58—H58	0.9500
C10—H10	0.9500	C59—C60	1.3642 (18)
C11—C12	1.418 (5)	C59—H59	0.9500
C11—H11	0.9500	C60—H60	0.9500
C12—C13	1.337 (4)	C61—C62	1.425 (4)
C12—H12	0.9500	C62—C63	1.407 (3)
C13—C14	1.338 (4)	C62—C64	1.444 (3)
C13—H13	0.9500	C63—H63	0.9500
C15—C16	1.363 (4)	C64—C65	1.506 (4)
C15—C20	1.386 (4)	C65—H65A	0.9800
C16—C17	1.299 (4)	C65—H65B	0.9800
C16—H16	0.9500	C65—H65C	0.9800
C17—C18	1.374 (4)	C66—C67	1.412 (4)
C17—H17	0.9500	C66—H66	0.9500
C18—C19	1.361 (4)	C67—C68	1.374 (4)
C18—H18	0.9500	C68—C69	1.421 (4)
C19—C20	1.312 (4)	C68—H68	0.9500
C19—H19	0.9500	C69—C74	1.406 (4)
C20—H20	0.9500	C69—C70	1.426 (4)
C21—C22	1.425 (3)	C70—C71	1.365 (4)
C22—C23	1.421 (4)	C70—H70	0.9500
C22—C24	1.434 (4)	C71—C72	1.403 (4)
C23—H23	0.9500	C71—H71	0.9500
C24—C25	1.497 (4)	C72—C73	1.369 (4)
C25—H25A	0.9800	C72—H72	0.9500
C25—H25B	0.9800	C73—C74	1.417 (4)
C25—H25C	0.9800	C73—H73	0.9500
C26—C27	1.380 (4)	C75—C80	1.377 (4)
C26—H26	0.9500	C75—C76	1.392 (4)
C27—C28	1.325 (3)	C76—C77	1.401 (4)
C28—C29	1.429 (4)	C76—H76	0.9500
C28—H28	0.9500	C77—C78	1.360 (4)
C29—C34	1.369 (4)	C77—H77	0.9500
C29—C30	1.375 (4)	C78—C79	1.384 (4)
C30—C31	1.381 (4)	C78—H78	0.9500
C30—H30	0.9500	C79—C80	1.393 (4)
C31—C32	1.357 (4)	C79—H79	0.9500
C31—H31	0.9500	C80—H80	0.9500
C32—C33	1.320 (4)	O5—C81	1.370 (4)
C32—H32	0.9500	O5—H5O	0.9055
C33—C34	1.437 (4)	C81—H81A	0.9800
C33—H33	0.9500	C81—H81B	0.9800
C35—C40	1.401 (4)	C81—H81C	0.9800
C35—C36	1.402 (4)	O6—C82	1.420 (3)
C36—C37	1.399 (4)	O6—H6O	0.9121
C36—H36	0.9500	C82—H82A	0.9800
C37—C38	1.379 (5)	C82—H82B	0.9800
C37—H37	0.9500	C82—H82C	0.9800
C38—C39	1.385 (5)	O7—C83	1.390 (3)
C38—H38	0.9500	O7—H7O	0.9094
C39—C40	1.390 (4)	C83—H83A	0.9800
C39—H39	0.9500	C83—H83B	0.9800
C40—H40	0.9500	C83—H83C	0.9800
Zn2—O3	1.8992 (19)	O7'—C83'	1.400 (3)
Zn2—N9	1.954 (2)	O7'—H7OA	0.8999
Zn2—O4	1.9616 (17)	C83'—H83D	0.9800
Zn2—N13	2.041 (2)	C83'—H83E	0.9800
O3—C41	1.282 (3)	C83'—H83F	0.9800
O4—C61	1.290 (3)	O8—C84	1.392 (3)
N9—C43	1.337 (3)	O8—H8O	0.9090
N9—C47	1.354 (3)	C84—H84A	0.9800
N10—C55	1.326 (3)	C84—H84B	0.9800
N10—C41	1.336 (3)	C84—H84C	0.9800
N10—N11	1.387 (3)	O9—C85	1.405 (4)
N11—C44	1.234 (3)	O9—H9O	0.9038
N12—C46	1.261 (3)	C85—H85A	0.9800
N12—C54	1.310 (3)	C85—H85B	0.9800
N13—C63	1.328 (3)	C85—H85C	0.9800
			
O1—Zn1—O2	121.00 (8)	N15—N14—C75	119.40 (19)
O1—Zn1—N1	94.83 (8)	C64—N15—N14	105.4 (2)
O2—Zn1—N1	111.47 (9)	C66—N16—C74	117.8 (2)
O1—Zn1—N5	112.58 (8)	O3—C41—N10	126.3 (2)
O2—Zn1—N5	99.04 (8)	O3—C41—C42	130.6 (2)
N1—Zn1—N5	119.45 (9)	N10—C41—C42	103.1 (2)
C1—O1—Zn1	120.56 (15)	C43—C42—C41	124.1 (2)
C21—O2—Zn1	117.75 (15)	C43—C42—C44	129.6 (2)
C3—N1—C7	117.4 (2)	C41—C42—C44	106.4 (2)
C3—N1—Zn1	123.56 (17)	C42—C43—N9	126.9 (2)
C7—N1—Zn1	118.95 (18)	C42—C43—H43	116.6
C1—N2—C15	126.9 (2)	N9—C43—H43	116.6
C1—N2—N3	114.0 (2)	N11—C44—C45	118.2 (2)
C15—N2—N3	118.8 (2)	N11—C44—C42	112.3 (2)
C4—N3—N2	104.2 (2)	C45—C44—C42	129.5 (2)
C6—N4—C14	114.4 (2)	C44—C45—H45A	109.5
C23—N5—C27	118.7 (2)	C44—C45—H45B	109.5
C23—N5—Zn1	121.08 (17)	H45A—C45—H45B	109.5
C27—N5—Zn1	119.84 (16)	C44—C45—H45C	109.5
C21—N6—N7	111.97 (19)	H45A—C45—H45C	109.5
C21—N6—C35	128.4 (2)	H45B—C45—H45C	109.5
N7—N6—C35	119.3 (2)	N12—C46—C47	126.7 (2)
C24—N7—N6	104.9 (2)	N12—C46—H46	116.6
C26—N8—C34	118.9 (2)	C47—C46—H46	116.6
O1—C1—N2	124.8 (2)	C48—C47—N9	123.2 (2)
O1—C1—C2	131.9 (2)	C48—C47—C46	118.8 (2)
N2—C1—C2	103.3 (2)	N9—C47—C46	117.9 (2)
C3—C2—C1	124.7 (3)	C47—C48—C49	116.6 (3)
C3—C2—C4	127.3 (2)	C47—C48—H48	121.7
C1—C2—C4	108.0 (2)	C49—C48—H48	121.7
N1—C3—C2	124.0 (2)	C48—C49—C50	119.3 (3)
N1—C3—H3	118.0	C48—C49—C54	120.4 (2)
C2—C3—H3	118.0	C50—C49—C54	120.3 (2)
N3—C4—C2	110.5 (2)	C51—C50—C49	116.6 (3)
N3—C4—C5	118.5 (2)	C51—C50—H50	121.7
C2—C4—C5	131.0 (2)	C49—C50—H50	121.7
C4—C5—H5A	109.5	C50—C51—C52	123.0 (3)
C4—C5—H5B	109.5	C50—C51—H51	118.5
H5A—C5—H5B	109.5	C52—C51—H51	118.5
C4—C5—H5C	109.5	C53—C52—C51	121.6 (3)
H5A—C5—H5C	109.5	C53—C52—H52	119.2
H5B—C5—H5C	109.5	C51—C52—H52	119.2
N4—C6—C7	125.0 (3)	C52—C53—C54	116.8 (3)
N4—C6—H6	117.5	C52—C53—H53	121.6
C7—C6—H6	117.5	C54—C53—H53	121.6
C8—C7—N1	121.6 (3)	N12—C54—C53	115.7 (3)
C8—C7—C6	121.1 (2)	N12—C54—C49	122.6 (2)
N1—C7—C6	117.3 (2)	C53—C54—C49	121.8 (2)
C7—C8—C9	116.3 (3)	N10—C55—C56	116.09 (19)
C7—C8—H8	121.9	N10—C55—C60	117.83 (19)
C9—C8—H8	121.9	C56—C55—C60	126.1 (2)
C8—C9—C10	119.9 (3)	C57—C56—C55	116.8 (2)
C8—C9—C14	119.3 (2)	C57—C56—H56	121.6
C10—C9—C14	120.8 (3)	C55—C56—H56	121.6
C11—C10—C9	117.4 (3)	C56—C57—C58	117.7 (2)
C11—C10—H10	121.3	C56—C57—H57	121.2
C9—C10—H10	121.3	C58—C57—H57	121.2
C10—C11—C12	121.5 (3)	C59—C58—C57	124.8 (2)
C10—C11—H11	119.3	C59—C58—H58	117.6
C12—C11—H11	119.3	C57—C58—H58	117.6
C13—C12—C11	122.7 (3)	C60—C59—C58	118.5 (2)
C13—C12—H12	118.7	C60—C59—H59	120.8
C11—C12—H12	118.7	C58—C59—H59	120.8
C12—C13—C14	116.7 (3)	C59—C60—C55	116.2 (2)
C12—C13—H13	121.7	C59—C60—H60	121.9
C14—C13—H13	121.7	C55—C60—H60	121.9
C13—C14—N4	115.0 (3)	O4—C61—N14	123.0 (2)
C13—C14—C9	120.9 (3)	O4—C61—C62	130.8 (2)
N4—C14—C9	124.0 (2)	N14—C61—C62	106.2 (2)
N2—C15—C16	116.3 (2)	C63—C62—C61	127.9 (2)
N2—C15—C20	120.4 (2)	C63—C62—C64	127.0 (3)
C16—C15—C20	123.4 (2)	C61—C62—C64	104.9 (2)
C17—C16—C15	117.7 (3)	N13—C63—C62	124.9 (3)
C17—C16—H16	121.2	N13—C63—H63	117.5
C15—C16—H16	121.2	C62—C63—H63	117.5
C16—C17—C18	119.6 (3)	N15—C64—C62	111.8 (2)
C16—C17—H17	120.2	N15—C64—C65	121.3 (2)
C18—C17—H17	120.2	C62—C64—C65	126.9 (2)
C19—C18—C17	122.9 (3)	C64—C65—H65A	109.5
C19—C18—H18	118.5	C64—C65—H65B	109.5
C17—C18—H18	118.5	H65A—C65—H65B	109.5
C20—C19—C18	118.2 (3)	C64—C65—H65C	109.5
C20—C19—H19	120.9	H65A—C65—H65C	109.5
C18—C19—H19	120.9	H65B—C65—H65C	109.5
C19—C20—C15	118.2 (3)	N16—C66—C67	124.0 (3)
C19—C20—H20	120.9	N16—C66—H66	118.0
C15—C20—H20	120.9	C67—C66—H66	118.0
O2—C21—N6	123.4 (2)	C68—C67—C66	118.0 (2)
O2—C21—C22	130.9 (2)	C68—C67—N13	125.5 (2)
N6—C21—C22	105.8 (2)	C66—C67—N13	116.5 (2)
C23—C22—C21	126.5 (2)	C67—C68—C69	120.2 (2)
C23—C22—C24	128.5 (2)	C67—C68—H68	119.9
C21—C22—C24	105.0 (2)	C69—C68—H68	119.9
N5—C23—C22	124.4 (2)	C74—C69—C68	117.2 (3)
N5—C23—H23	117.8	C74—C69—C70	119.5 (2)
C22—C23—H23	117.8	C68—C69—C70	123.3 (2)
N7—C24—C22	112.4 (2)	C71—C70—C69	120.9 (3)
N7—C24—C25	120.8 (2)	C71—C70—H70	119.6
C22—C24—C25	126.8 (2)	C69—C70—H70	119.6
C24—C25—H25A	109.5	C70—C71—C72	119.5 (3)
C24—C25—H25B	109.5	C70—C71—H71	120.3
H25A—C25—H25B	109.5	C72—C71—H71	120.3
C24—C25—H25C	109.5	C73—C72—C71	120.9 (3)
H25A—C25—H25C	109.5	C73—C72—H72	119.5
H25B—C25—H25C	109.5	C71—C72—H72	119.5
N8—C26—C27	125.2 (2)	C72—C73—C74	120.9 (3)
N8—C26—H26	117.4	C72—C73—H73	119.5
C27—C26—H26	117.4	C74—C73—H73	119.5
C28—C27—C26	116.4 (3)	N16—C74—C69	122.6 (2)
C28—C27—N5	123.9 (2)	N16—C74—C73	119.1 (2)
C26—C27—N5	119.6 (2)	C69—C74—C73	118.3 (3)
C27—C28—C29	120.1 (3)	C80—C75—C76	119.6 (3)
C27—C28—H28	119.9	C80—C75—N14	120.7 (2)
C29—C28—H28	119.9	C76—C75—N14	119.7 (2)
C34—C29—C30	116.7 (3)	C75—C76—C77	119.6 (3)
C34—C29—C28	119.5 (2)	C75—C76—H76	120.2
C30—C29—C28	123.7 (3)	C77—C76—H76	120.2
C29—C30—C31	121.7 (3)	C78—C77—C76	121.0 (3)
C29—C30—H30	119.1	C78—C77—H77	119.5
C31—C30—H30	119.1	C76—C77—H77	119.5
C32—C31—C30	121.6 (3)	C77—C78—C79	119.0 (3)
C32—C31—H31	119.2	C77—C78—H78	120.5
C30—C31—H31	119.2	C79—C78—H78	120.5
C33—C32—C31	118.3 (3)	C78—C79—C80	121.2 (3)
C33—C32—H32	120.9	C78—C79—H79	119.4
C31—C32—H32	120.9	C80—C79—H79	119.4
C32—C33—C34	121.7 (3)	C75—C80—C79	119.6 (2)
C32—C33—H33	119.2	C75—C80—H80	120.2
C34—C33—H33	119.2	C79—C80—H80	120.2
N8—C34—C29	119.8 (2)	C81—O5—H5O	110.1
N8—C34—C33	120.1 (2)	O5—C81—H81A	109.5
C29—C34—C33	120.0 (2)	O5—C81—H81B	109.5
C40—C35—C36	120.6 (2)	H81A—C81—H81B	109.5
C40—C35—N6	119.6 (3)	O5—C81—H81C	109.5
C36—C35—N6	119.8 (2)	H81A—C81—H81C	109.5
C37—C36—C35	119.6 (3)	H81B—C81—H81C	109.5
C37—C36—H36	120.2	C82—O6—H6O	109.7
C35—C36—H36	120.2	O6—C82—H82A	109.5
C38—C37—C36	119.8 (3)	O6—C82—H82B	109.5
C38—C37—H37	120.1	H82A—C82—H82B	109.5
C36—C37—H37	120.1	O6—C82—H82C	109.5
C37—C38—C39	120.3 (3)	H82A—C82—H82C	109.5
C37—C38—H38	119.8	H82B—C82—H82C	109.5
C39—C38—H38	119.8	C83—O7—H7O	109.4
C38—C39—C40	121.4 (3)	O7—C83—H83A	109.5
C38—C39—H39	119.3	O7—C83—H83B	109.5
C40—C39—H39	119.3	H83A—C83—H83B	109.5
C39—C40—C35	118.3 (3)	O7—C83—H83C	109.5
C39—C40—H40	120.8	H83A—C83—H83C	109.5
C35—C40—H40	120.8	H83B—C83—H83C	109.5
O3—Zn2—N9	95.73 (8)	C83'—O7'—H7OA	109.6
O3—Zn2—O4	118.13 (9)	O7'—C83'—H83D	109.5
N9—Zn2—O4	109.94 (8)	O7'—C83'—H83E	109.5
O3—Zn2—N13	115.06 (8)	H83D—C83'—H83E	109.5
N9—Zn2—N13	118.36 (10)	O7'—C83'—H83F	109.5
O4—Zn2—N13	100.62 (8)	H83D—C83'—H83F	109.5
C41—O3—Zn2	121.49 (16)	H83E—C83'—H83F	109.5
C61—O4—Zn2	116.80 (16)	C84—O8—H8O	109.7
C43—N9—C47	120.6 (2)	O8—C84—H84A	109.5
C43—N9—Zn2	121.09 (17)	O8—C84—H84B	109.5
C47—N9—Zn2	118.28 (17)	H84A—C84—H84B	109.5
C55—N10—C41	127.2 (2)	O8—C84—H84C	109.5
C55—N10—N11	117.63 (19)	H84A—C84—H84C	109.5
C41—N10—N11	115.1 (2)	H84B—C84—H84C	109.5
C44—N11—N10	103.2 (2)	C85—O9—H9O	109.3
C46—N12—C54	114.7 (2)	O9—C85—H85A	109.5
C63—N13—C67	117.6 (2)	O9—C85—H85B	109.5
C63—N13—Zn2	118.75 (18)	H85A—C85—H85B	109.5
C67—N13—Zn2	123.63 (17)	O9—C85—H85C	109.5
C61—N14—N15	111.7 (2)	H85A—C85—H85C	109.5
C61—N14—C75	128.9 (2)	H85B—C85—H85C	109.5
			
O2—Zn1—O1—C1	111.56 (19)	N9—Zn2—O3—C41	−2.09 (19)
N1—Zn1—O1—C1	−7.08 (19)	O4—Zn2—O3—C41	114.16 (19)
N5—Zn1—O1—C1	−131.79 (18)	N13—Zn2—O3—C41	−127.12 (19)
C1—N2—N3—C4	−0.8 (3)	C55—N10—N11—C44	−179.1 (2)
C15—N2—N3—C4	173.0 (2)	C41—N10—N11—C44	1.3 (3)
C21—N6—N7—C24	1.7 (3)	C61—N14—N15—C64	0.4 (3)
C35—N6—N7—C24	−172.4 (2)	C75—N14—N15—C64	180.0 (2)
Zn1—O1—C1—N2	−171.44 (18)	Zn2—O3—C41—N10	−179.97 (19)
Zn1—O1—C1—C2	6.6 (4)	Zn2—O3—C41—C42	−0.4 (4)
C15—N2—C1—O1	6.3 (4)	C55—N10—C41—O3	−1.5 (4)
N3—N2—C1—O1	179.5 (2)	N11—N10—C41—O3	178.0 (2)
C15—N2—C1—C2	−172.3 (2)	C55—N10—C41—C42	178.8 (2)
N3—N2—C1—C2	1.0 (3)	N11—N10—C41—C42	−1.7 (3)
O1—C1—C2—C3	−2.3 (5)	O3—C41—C42—C43	2.3 (4)
N2—C1—C2—C3	176.1 (2)	N10—C41—C42—C43	−178.0 (2)
O1—C1—C2—C4	−179.2 (3)	O3—C41—C42—C44	−178.3 (3)
N2—C1—C2—C4	−0.8 (3)	N10—C41—C42—C44	1.3 (3)
C7—N1—C3—C2	178.5 (2)	C41—C42—C43—N9	−0.4 (4)
Zn1—N1—C3—C2	−4.2 (3)	C44—C42—C43—N9	−179.6 (2)
C1—C2—C3—N1	0.8 (4)	C47—N9—C43—C42	179.6 (2)
C4—C2—C3—N1	177.1 (2)	Zn2—N9—C43—C42	−2.9 (4)
N2—N3—C4—C2	0.2 (3)	N10—N11—C44—C45	−179.9 (2)
N2—N3—C4—C5	−179.7 (2)	N10—N11—C44—C42	−0.3 (3)
C3—C2—C4—N3	−176.4 (2)	C43—C42—C44—N11	178.6 (3)
C1—C2—C4—N3	0.4 (3)	C41—C42—C44—N11	−0.7 (3)
C3—C2—C4—C5	3.4 (4)	C43—C42—C44—C45	−1.9 (5)
C1—C2—C4—C5	−179.8 (3)	C41—C42—C44—C45	178.8 (3)
C14—N4—C6—C7	−1.7 (4)	C54—N12—C46—C47	0.7 (4)
C3—N1—C7—C8	−36.2 (3)	C43—N9—C47—C48	5.3 (4)
Zn1—N1—C7—C8	146.4 (2)	Zn2—N9—C47—C48	−172.3 (2)
C3—N1—C7—C6	144.8 (2)	C43—N9—C47—C46	−173.6 (2)
Zn1—N1—C7—C6	−32.7 (3)	Zn2—N9—C47—C46	8.7 (3)
N4—C6—C7—C8	1.3 (4)	N12—C46—C47—C48	−3.2 (4)
N4—C6—C7—N1	−179.6 (2)	N12—C46—C47—N9	175.8 (2)
N1—C7—C8—C9	−179.1 (2)	N9—C47—C48—C49	−175.5 (2)
C6—C7—C8—C9	−0.1 (4)	C46—C47—C48—C49	3.4 (4)
C7—C8—C9—C10	177.5 (2)	C47—C48—C49—C50	177.6 (2)
C7—C8—C9—C14	−0.5 (4)	C47—C48—C49—C54	−1.7 (4)
C8—C9—C10—C11	−177.0 (3)	C48—C49—C50—C51	−178.0 (2)
C14—C9—C10—C11	0.9 (4)	C54—C49—C50—C51	1.3 (4)
C9—C10—C11—C12	−1.1 (4)	C49—C50—C51—C52	−1.2 (4)
C10—C11—C12—C13	0.4 (5)	C50—C51—C52—C53	0.1 (4)
C11—C12—C13—C14	0.6 (4)	C51—C52—C53—C54	0.9 (4)
C12—C13—C14—N4	177.4 (2)	C46—N12—C54—C53	−178.5 (2)
C12—C13—C14—C9	−0.8 (4)	C46—N12—C54—C49	1.2 (4)
C6—N4—C14—C13	−177.1 (2)	C52—C53—C54—N12	179.0 (2)
C6—N4—C14—C9	1.0 (4)	C52—C53—C54—C49	−0.8 (4)
C8—C9—C14—C13	178.0 (2)	C48—C49—C54—N12	−0.7 (4)
C10—C9—C14—C13	0.1 (4)	C50—C49—C54—N12	179.9 (2)
C8—C9—C14—N4	0.0 (4)	C48—C49—C54—C53	179.0 (2)
C10—C9—C14—N4	−177.9 (2)	C50—C49—C54—C53	−0.4 (4)
C1—N2—C15—C16	159.1 (2)	C41—N10—C55—C56	165.9 (2)
N3—N2—C15—C16	−13.9 (3)	N11—N10—C55—C56	−13.6 (3)
C1—N2—C15—C20	−20.9 (4)	C41—N10—C55—C60	−14.8 (4)
N3—N2—C15—C20	166.1 (2)	N11—N10—C55—C60	165.7 (2)
N2—C15—C16—C17	−179.3 (3)	N10—C55—C56—C57	−178.7 (2)
C20—C15—C16—C17	0.7 (4)	C60—C55—C56—C57	2.1 (4)
C15—C16—C17—C18	0.5 (4)	C55—C56—C57—C58	−1.6 (4)
C16—C17—C18—C19	−1.0 (5)	C56—C57—C58—C59	0.5 (4)
C17—C18—C19—C20	0.2 (5)	C57—C58—C59—C60	0.3 (4)
C18—C19—C20—C15	0.9 (5)	C58—C59—C60—C55	0.0 (4)
N2—C15—C20—C19	178.6 (3)	N10—C55—C60—C59	179.5 (2)
C16—C15—C20—C19	−1.4 (5)	C56—C55—C60—C59	−1.3 (4)
Zn1—O2—C21—N6	173.05 (17)	Zn2—O4—C61—N14	179.41 (17)
Zn1—O2—C21—C22	−7.7 (3)	Zn2—O4—C61—C62	−2.6 (3)
N7—N6—C21—O2	176.7 (2)	N15—N14—C61—O4	178.0 (2)
C35—N6—C21—O2	−9.9 (4)	C75—N14—C61—O4	−1.6 (4)
N7—N6—C21—C22	−2.7 (3)	N15—N14—C61—C62	−0.5 (3)
C35—N6—C21—C22	170.7 (2)	C75—N14—C61—C62	−180.0 (2)
O2—C21—C22—C23	5.9 (4)	O4—C61—C62—C63	−1.6 (4)
N6—C21—C22—C23	−174.8 (2)	N14—C61—C62—C63	176.6 (2)
O2—C21—C22—C24	−176.9 (2)	O4—C61—C62—C64	−177.9 (2)
N6—C21—C22—C24	2.5 (3)	N14—C61—C62—C64	0.3 (3)
C27—N5—C23—C22	173.7 (2)	C67—N13—C63—C62	−179.3 (2)
Zn1—N5—C23—C22	1.0 (3)	Zn2—N13—C63—C62	−1.3 (3)
C21—C22—C23—N5	−1.7 (4)	C61—C62—C63—N13	3.9 (4)
C24—C22—C23—N5	−178.3 (2)	C64—C62—C63—N13	179.4 (2)
N6—N7—C24—C22	0.0 (3)	N14—N15—C64—C62	−0.2 (3)
N6—N7—C24—C25	−179.8 (2)	N14—N15—C64—C65	−179.5 (2)
C23—C22—C24—N7	175.6 (2)	C63—C62—C64—N15	−176.4 (2)
C21—C22—C24—N7	−1.6 (3)	C61—C62—C64—N15	−0.1 (3)
C23—C22—C24—C25	−4.6 (4)	C63—C62—C64—C65	2.8 (4)
C21—C22—C24—C25	178.2 (2)	C61—C62—C64—C65	179.2 (2)
C34—N8—C26—C27	−1.1 (4)	C74—N16—C66—C67	1.0 (4)
N8—C26—C27—C28	−0.1 (4)	N16—C66—C67—C68	4.0 (4)
N8—C26—C27—N5	176.4 (2)	N16—C66—C67—N13	−176.0 (2)
C23—N5—C27—C28	−48.8 (3)	C63—N13—C67—C68	−26.4 (3)
Zn1—N5—C27—C28	124.0 (2)	Zn2—N13—C67—C68	155.7 (2)
C23—N5—C27—C26	134.9 (2)	C63—N13—C67—C66	153.6 (2)
Zn1—N5—C27—C26	−52.3 (3)	Zn2—N13—C67—C66	−24.2 (3)
C26—C27—C28—C29	1.6 (4)	C66—C67—C68—C69	−5.4 (4)
N5—C27—C28—C29	−174.7 (2)	N13—C67—C68—C69	174.6 (2)
C27—C28—C29—C34	−2.1 (4)	C67—C68—C69—C74	2.0 (3)
C27—C28—C29—C30	174.6 (3)	C67—C68—C69—C70	−175.7 (2)
C34—C29—C30—C31	1.3 (4)	C74—C69—C70—C71	−0.8 (4)
C28—C29—C30—C31	−175.4 (3)	C68—C69—C70—C71	176.9 (2)
C29—C30—C31—C32	−1.5 (5)	C69—C70—C71—C72	0.5 (4)
C30—C31—C32—C33	−0.3 (4)	C70—C71—C72—C73	0.0 (4)
C31—C32—C33—C34	2.1 (4)	C71—C72—C73—C74	−0.1 (4)
C26—N8—C34—C29	0.6 (4)	C66—N16—C74—C69	−4.7 (4)
C26—N8—C34—C33	−175.8 (2)	C66—N16—C74—C73	175.7 (2)
C30—C29—C34—N8	−176.0 (2)	C68—C69—C74—N16	3.3 (4)
C28—C29—C34—N8	0.9 (4)	C70—C69—C74—N16	−179.0 (2)
C30—C29—C34—C33	0.5 (4)	C68—C69—C74—C73	−177.2 (2)
C28—C29—C34—C33	177.3 (2)	C70—C69—C74—C73	0.6 (3)
C32—C33—C34—N8	174.2 (2)	C72—C73—C74—N16	179.4 (2)
C32—C33—C34—C29	−2.2 (4)	C72—C73—C74—C69	−0.1 (4)
C21—N6—C35—C40	−20.8 (4)	C61—N14—C75—C80	−28.5 (4)
N7—N6—C35—C40	152.1 (2)	N15—N14—C75—C80	152.0 (2)
C21—N6—C35—C36	160.8 (2)	C61—N14—C75—C76	153.6 (2)
N7—N6—C35—C36	−26.2 (3)	N15—N14—C75—C76	−25.9 (3)
C40—C35—C36—C37	−0.7 (4)	C80—C75—C76—C77	−0.7 (4)
N6—C35—C36—C37	177.7 (2)	N14—C75—C76—C77	177.3 (2)
C35—C36—C37—C38	1.0 (4)	C75—C76—C77—C78	0.9 (4)
C36—C37—C38—C39	−0.5 (4)	C76—C77—C78—C79	−0.7 (4)
C37—C38—C39—C40	−0.3 (4)	C77—C78—C79—C80	0.3 (4)
C38—C39—C40—C35	0.7 (4)	C76—C75—C80—C79	0.3 (4)
C36—C35—C40—C39	−0.2 (4)	N14—C75—C80—C79	−177.7 (2)
N6—C35—C40—C39	−178.6 (2)	C78—C79—C80—C75	−0.1 (4)

### Hydrogen-bond geometry (Å, º)

**Table:**

*D*—H···*A*	*D*—H	H···*A*	*D*···*A*	*D*—H···*A*
C5—H5*A*···O5^i^	0.98	2.43	3.312 (4)	149
C6—H6···O2	0.95	2.25	3.148 (4)	157
C23—H23···O6^ii^	0.95	2.31	3.260 (3)	179
C46—H46···N13	0.95	2.56	3.368 (3)	144
C66—H66···O3	0.95	2.46	3.344 (4)	155
C68—H68···O9^iii^	0.95	2.40	3.334 (4)	170
O5—H5*O*···N3^iv^	0.91	2.10	2.977 (4)	164
O6—H6*O*···N4	0.91	1.92	2.830 (3)	175
O7—H7*O*···N8	0.91	2.10	2.993 (4)	168
O7′—H7*OA*···N8	0.90	1.89	2.794 (10)	179
O8—H8*O*···N12^v^	0.91	1.91	2.816 (3)	173
O9—H9*O*···O8	0.90	1.73	2.622 (3)	170
C85—H85*C*···O9^vi^	0.98	2.46	3.340 (4)	150

Symmetry codes: (i) *x*, *y*, *z*+1; (ii) −*x*+1, −*y*+1, −*z*+1; (iii) *x*−1, *y*, *z*; (iv) *x*, *y*, *z*−1; (v) *x*+1, *y*, *z*; (vi) −*x*+2, −*y*, −*z*.
